# Yeast mutation rates in alternative carbon sources reflect the influence of reactive oxygen species

**DOI:** 10.17912/micropub.biology.001429

**Published:** 2025-02-19

**Authors:** Keerthana Thota, Jacob D. Fredette-Roman, Nathaniel P. Sharp

**Affiliations:** 1 Department of Genetics , University of Wisconsin–Madison, Madison, Wisconsin, United States

## Abstract

Environmental conditions can influence mutation rates, but the reasons are often unclear. Budding yeast can utilize many carbon sources, with variation in the degree of fermentation versus respiration. Since aerobic respiration produces mutagenic reactive oxygen species, we hypothesized that yeast grown in media promoting aerobic respiration would show higher mutation rates. We found significant differences across five media types, with the highest mutation rate in pyruvate and the lowest in glucose. However, mutation rates responded to respiration rate in a nonlinear fashion, suggesting that the degree of respiration in a given environment is only partly predictive of mutation rate.

**Figure 1. Mutation rates in yeast growing with alternative carbon sources.**
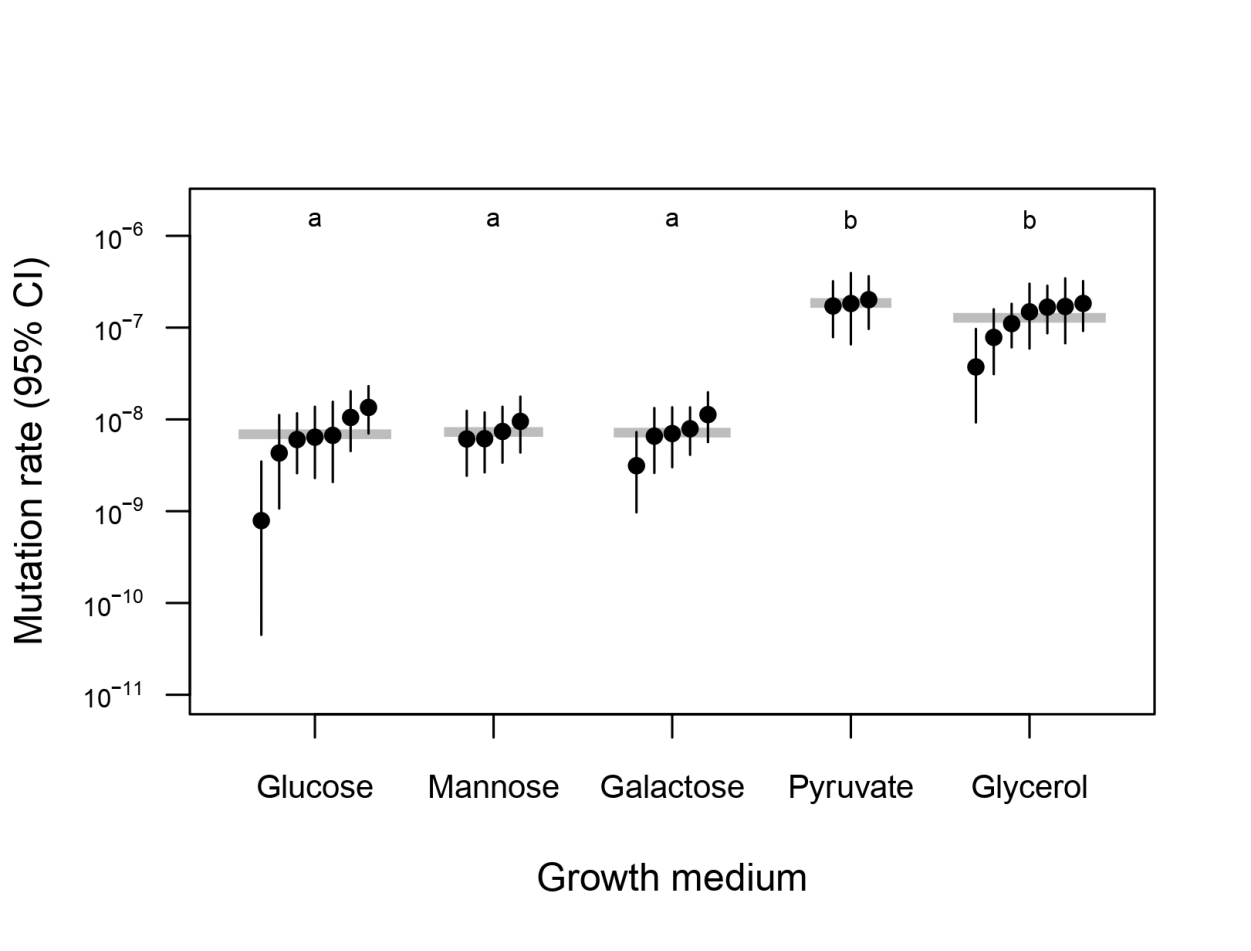
Mutation rates are shown on a log scale with 95% confidence intervals. Each point represents one fluctuation test involving 72 replicate populations, in ascending order of mutation rate within each group. Horizontal grey bars indicate group means. Groups with significantly different mutation rates based on Tukey HSD tests are labeled with different letters.

## Description

There is evidence that mutation rates can differ between environments, but the mechanistic causes of this variation are not always clear. For example, in the budding yeast *Saccharomyces cerevisiae* , point mutation rates were found to vary 3.6-fold across seven media environments, with generally higher mutation rates in environments where growth was slower (Liu and Zhang 2019) , in accordance with evidence for stress-associated mutagenesis in eukaryotes more generally (Goho and Bell 2000; Lang 2007; Lamb et al. 2008; Agrawal and Wang 2008; Matsuba et al. 2012; Shor et al. 2013; Jiang et al. 2014; Sharp and Agrawal 2012; Sharp and Agrawal 2016). Our goal was to consider yeast mutation rates in relation to a particular dimension of environmental variability: the extent to which a given carbon source is metabolized through fermentation versus respiration.

In *S. cerevisiae* , fermentation is the major pathway for energy production, even when there is oxygen present. During fermentation, yeast cells convert sugars into ethanol and CO _2_ . When the sugar levels drop sufficiently, ethanol produced during fermentation is used as a carbon source, shifting the primary energy pathway to respiration (Käppeli, 1987) . In the presence of oxygen, aerobic respiration converts sugar into CO _2_ and water. Depending on the carbon source, *S. cerevisiae* display various degrees of respiration (Fendt and Sauer 2010) . Laboratory yeast are commonly grown in yeast-extract peptone dextrose (YPD), which contains a common form of glucose as the carbon source, allowing for a high level of fermentation. In contrast, glycerol and pyruvate are non-fermentable carbon sources on which yeast cells can only grow by aerobic respiration (Schüller 2003, Fendt and Sauer 2010) . Galactose is presumed to cause simultaneous fermentation and respiration, and mannose causes decreased carbon catabolite repression (CCR); CCR is what normally causes *S. cerevisiae* to prefer glucose to alternative carbon sources, and mannose’s CCR response is weaker than glucose, shifting cells slightly towards respiration (Fendt and Sauer 2010) .

In short, glucose, mannose, galactose, glycerol and pyruvate are metabolized differently. Some of these carbon sources will lead to a shift from fermentation to respiration sooner than others, but *S. cerevisiae* will always prefer to undergo fermentation if a fermentable carbon source is available (Gancedo 1998) . Aerobic respiration produces mutagenic reactive oxygen species (ROS), which can damage DNA, increasing the potential for mutation (Martin and Palumbi 1993) . We therefore predicted that yeast cultured in media promoting more aerobic respiration would exhibit increased mutation rates.

To test this prediction, we performed a series of fluctuation tests (Lang and Murray 2008, Dunham et al. 2015) to measure phenotypic mutation rates in 1872 yeast populations growing in the five different carbon sources described above ( Fig. 1 ). This is a common approach that reflects the rate of mutation to null alleles at a counter-selectable marker locus. We detected significant variation in mutation rate across carbon sources (analysis of variance: *F*_4,21_ = 35.53, *P* = 4.55 × 10 ^–9^ ). Relative to glucose, the mutation rates of yeast grown with the non-fermentable carbon sources glycerol and pyruvate were elevated by 18.6-fold and 26.9-fold, respectively. In mannose and galactose, where intermediate respiration rates are expected, we found that mutation rates were elevated by 1.06-fold and 1.04-fold, respectively, relative to glucose. Based on post-hoc Tukey HSD testing, the five media types form two significantly different groups: glucose, mannose and galactose with lower mutation rates, and pyruvate and glycerol with higher mutation rates.

While our data support the idea that respiration is associated with higher mutation rates, this relationship does not appear to be linear. Indeed, we only detected increased mutation rates in the context of obligate respiration, and not in media where intermediate rates of respiration are expected. Future work establishing oxygen consumption rates and levels of ROS for cells growing on alternative carbon sources would further clarify whether these features of an environment are predictive of mutation rates.

## Methods

*Yeast strains and growth media*

We conducted all experiments with a haploid (euploid) version of the strain YPS1009 (Hose et al. 2020) at 30 C. Each type of growth media consisted of 20 g/L of sugar (mannose, galactose, pyruvate, glucose or glycerol), 10 g/L of yeast extract, and 20 g/L of peptone. We included ampicillin (40 mg/L) to prevent bacterial contamination; any experimental blocks showing signs of contamination were excluded from our dataset. For fluctuation tests, we prepared synthetic complete uracil dropout media (SC–Ura) and 5-fluoro-orotic acid media (5-FOA) as described in Dunham et al. (2015).

*Fluctuation tests*

We used a standard fluctuation test approach described previously, where mutations disrupting *URA3* or *URA5* are selectable on 5-FOA (Lang 2008, Lang 2018) . Yeast only grow on 5-FOA when there is a loss of function at the URA3 gene, which converts 5-FOA into a toxic inhibitor of thymidylate synthase. For each block of fluctuation tests we took yeast from frozen stock, streaked onto YPD plates, and grew them for three days. We then picked a single colony to inoculate 2 mL of SC-Ura to select against pre-existing mutations, incubated overnight, and then performed serial dilution into each type of focal media, with a final dilution factor of 1:10,000. The fluctuation test approach requires that mutant colonies appear in at least some replicate populations, but too many mutations is also not ideal due to the appearance of “jackpot” cases where mutant colonies are too numerous to count. We therefore grew populations in different volumes for different media environments based on preliminary assessments of growth rates and mutation rates. Specifically, we used 200 µL for glycerol, 50 µL for pyruvate, and 100 µL for glucose, galactose, and mannose, with populations growing in 96-well plates. We sealed the plate with a breathable seal and incubated it for two days. We standardized culture volumes just prior to plating by adding 100 µL of sorbitol to each well of the glucose, galactose, and mannose replicates, and 150 µL of sorbitol to the pyruvate replicates. For each 96-well plate, we transferred cultures from a random set of 24 wells into a test tube for cell counting and measurement of culture volume, and used this information to calculate the number of cells per population. We transferred the remaining 72 cultures from each 96-well plate onto 5-FOA plates, with 8 spots per plate. To facilitate liquid absorption we used 5-FOA plates that were pre-dried for one day in a 30 C incubator (Lang 2018) . After allowing the liquid to absorb, we inverted the plates and incubated them for three days. We then counted colonies in each spot using a dissection microscope.

*Calculating Mutation rates and significance*

We used the *RSalvador* package (Zheng 2021, Zheng 2022) in *R* (R Core Team, 2023) to determine mutation rates and 95% confidence intervals based on mutant colony counts and population sizes. We used ANOVA to test for variation in mutation rates among media types, followed by Tukey HSD post-hoc comparisons, accounting for multiple testing.
